# Imaging Ligand‐Receptor Interactions at Single‐Protein Resolution with DNA‐PAINT

**DOI:** 10.1002/smtd.202401799

**Published:** 2025-04-03

**Authors:** Monique Honsa, Isabelle Pachmayr, Larissa Heinze, Levent Bas, Luciano A. Masullo, Jisoo Kwon, Ana Perovic, Brenda Schulman, Ralf Jungmann

**Affiliations:** ^1^ Max Planck Institute of Biochemistry Am Klopferspitz 18 82152 Planegg Germany; ^2^ Faculty of Physics and Center for Nanoscience Ludwig Maximilian University Geschwister‐Scholl‐Platz 1 80539 Munich Germany

**Keywords:** DNA‐PAINT, EGFR, ligand‐receptor interactions, membrane receptors, single‐molecule imaging, super‐resolution imaging

## Abstract

Ligand‐receptor interactions are critical for cell communication, with membrane receptors such as the Epidermal Growth Factor Receptor (EGFR) mediating responses to external signals. Super‐resolution microscopy techniques in principle allow the visualization of these interactions at single‐molecule resolution. While DNA‐Points Accumulation for Imaging in Nanoscale Topography (DNA‐PAINT) super‐resolution microscopy has been successfully used to image receptors, specific labeling of cognate ligands, such as EGF, with DNA has remained challenging. Here, an approach to label and image the small extracellular ligand EGF using site‐specific tagging and DNA modification is presented. Functional, site‐specifically tagged EGF constructs, including DNA‐conjugated and ALFA‐tagged EGF, are generated. When compared to the native ligand, only the ALFA‐tagged EGF maintains full functionality such as efficient EGFR clustering and internalization, while the DNA‐conjugated EGF exhibits reduced EGFR oligomerization. 3D DNA‐PAINT imaging of the ALFA‐tagged EGF, when bound to EGFR, reveals spatial arrangements of EGF‐EGFR complexes and captures different stages of receptor internalization. The labeling approach enables precise visualization of ligand‐receptor interactions at high resolution and, in principle, can be extended to other ligand‐receptor systems.

## Graphical abstract

A labeling approach for small ligands in DNA‐PAINT super‐resolution microscopy preserving ligand functionality is demonstrated. This method enables the study of ligand‐receptor interactions at single‐protein resolution providing detailed insights into their spatial arrangement and interactions. Here, the interaction of the Epidermal Growth Factor (EGF) and its receptor (EGFR) as a model ligand‐receptor system is specifically studied.

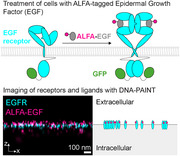

## 1 Introduction

Ligand‐receptor interactions play a major role in intercellular communication and decision‐making, with membrane receptors acting as key mediators to external signals.^[^ ^1^ ^]^ Ligands binding to these receptors are the primary source by which cells interpret and respond to their environment. This binding event triggers a cascade of downstream signaling, often regulated by other small proteins, that directs crucial cellular processes such as growth, differentiation, and survival.^[^ ^1^ ^]^ Understanding these interactions at the molecular level is essential, as ligand‐receptor interactions are fundamental for numerous biological functions and disease mechanisms.

To date, quantitatively studying these systems with single‐protein resolution is challenging, which limits our ability to fully understand their interactions and downstream effects on signaling pathways. Super‐resolution fluorescence microscopy methods allow for sub‐20 nm resolution imaging in situ and have enabled novel scientific discoveries in the last 20 years.^[^ ^2^ ^]^ One way to achieve super‐resolution is single‐molecule localization microscopy (SMLM).^[^ ^3^, ^4^, ^5^, ^6^ ^]^ In this method, individual and well‐separated fluorophores are imaged as they switch on and off stochastically over the sample. The super‐resolved image is reconstructed using the positions of each measured fluorophore, which are obtained from fits of their individual images to the point spread function of the microscope. DNA‐Points Accumulation for Imaging in Nanoscale Topography (DNA‐PAINT) is an SMLM method in which the temporal separation of emitters is achieved by transient binding of dye‐labeled imager strands (imager) to complementary DNA‐docking strands (binding site) that are immobilized on the targets of interest in the sample.^[^ ^7^, ^8^ ^]^

DNA‐PAINT has several advantages over other SMLM approaches: 1) since the ON/OFF switching mechanism is decoupled from the photophysics of the fluorescent dye, the brightest fluorophores available can be used, yielding higher signal‐to‐noise ratio, which in turn leads to improved localization precisions^[^ ^9^ ^]^ (up to 2–3 nm in cells), 2) repetitive and predictable sampling of the targets is achieved as a virtually unlimited pool of imager strands will transiently bind to the docking sites,^[^ ^10^ ^]^ 3) unlimited multiplexing can be achieved by using different DNA orthogonal sequences.^[^ ^11^, ^12^ ^]^ Therefore, DNA‐PAINT achieves a high‐fidelity, multi‐channel, 3D single‐protein resolution in whole intact cells (Fields of view ≈100 × 100 µm2). Furthermore, resolution can be extended to the Ångström level by using the recently developed Resolution Enhancement by Sequential Imaging (RESI) method.^[^ ^13^ ^]^ Given these advantages, DNA‐PAINT emerges as one of the methods of choice to directly image and quantify oligomerization and molecular organization of receptors and ligands in situ.

To robustly visualize membrane receptors with DNA‐PAINT, efficient labeling of the targets of interest with DNA strands is required. Standard protein labeling approaches for DNA‐PAINT include the use of primary antibodies with DNA‐labeled secondary probes^[^ ^14^ ^]^ as well as direct immunolabeling with DNA‐conjugated affibodies,^[^ ^15^ ^]^ aptamers^[^ ^16^ ^]^ and nanobodies.^[^ ^9^, ^14^ ^]^ While antibodies are the most widely available labeling reagents, their relatively large size (150 kDa) limits the positional accuracy in DNA‐PAINT measurements.^[^ ^17^ ^]^ Affibodies, aptamers, and nanobodies are smaller, however, they are often not readily available for specific targets of interest. To address this issue in experiments requiring the simultaneous targeting of only a few protein species, one effective approach is to use genetically encoded tags, such as Green Fluorescent Protein (GFP)^[^ ^18^ ^]^ or the ALFA tag,^[^ ^19^ ^]^ which have readily available cognate nanobody binders. DNA‐PAINT and RESI imaging of membrane receptors at single‐protein resolution have successfully been demonstrated in the past.^[^ ^13^ ^]^

However, there are currently no optimized methods to specifically label extracellular ligands for efficient DNA‐PAINT imaging while preserving their biological function. To demonstrate a universally applicable method for labeling and imaging ligands, we chose the well‐known and extensively studied EGF‐EGFR ligand‐receptor system.^[^ ^20^ ^]^ EGFR is one of four members of the EGFR receptor family, all of which play a crucial role in regulating cell proliferation, differentiation, and migration.^[^ ^21^, ^22^ ^]^ Additionally, EGFR family members are often mutated or dysregulated in cancers, such as those affecting the breast, lung, brain, and gastrointestinal tract.^[^ ^21^, ^22^ ^]^ As a result, drugs that modulate EGFR expression levels or EGFR activation—by targeting the extracellular or intracellular domains, respectively—are utilized in cancer therapy.

On a molecular level, EGFR activation is triggered by EGF‐induced structural alterations and the formation of an EGFR‐EGFR dimer interface, as shown by X‐ray crystallography.^[^ ^23^ ^]^ This leads to tyrosine autophosphorylation within the EGFR dimer's intracellular kinase domains.^[^ ^24^ ^]^ Understanding these interactions at the molecular level is crucial not only for basic biological insights into how cells communicate and respond to external signals but also for informing the design of targeted therapies that can more precisely modulate these pathways. Improved molecular‐level insights could therefore drive the development of more effective cancer treatments.

To date, direct stochastic optical reconstruction microscopy imaging showed EGF and EGFR co‐clustering, with resolution limited to ≈20 nm.^[^ ^25^ ^]^ Higher spatial resolutions were reached with Fluorescence resonance energy transfer and Fluorophore localization imaging with photobleaching studies of dye‐labeled EGF, indirectly measuring ≈12 nm EGF‐to‐EGF distances in cells.^[^ ^26^, ^27^ ^]^ However, these approaches do not allow for direct imaging of EGF‐to‐EGF distances with sub‐5 nm resolution and are mostly limited to single‐target imaging.^[^ ^28^, ^29^ ^]^

Determining the organization of EGF‐EGFR in the activated state necessitates multiplexed imaging of both EGFR and EGF at sub‐20 nm resolution, as the expected EGF‐to‐EGF distance when bound to an EGFR dimer is ≈11 nm. With the advancement of sub‐5‐nm imaging as in RESI, we can now visualize the molecular arrangement of EGF when bound to EGFR in a cellular context, in 3D. This capability promises to deepen our understanding of receptor‐ligand interactions with an unparalleled level of detail, potentially paving the way for breakthroughs in therapeutic targeting. However, the imaging of EGF with DNA‐PAINT has been limited by a lack of efficient labeling strategies. In this study, we thus developed a functional, site‐specifically tagged human EGF ligand with high yields by utilizing two tags that enhance EGF expression and folding. We assess both the functionality and site‐specific addressability of these ligands for DNA‐PAINT imaging and propose a broadly applicable labeling method for small extracellular ligands.

## 2 Results

**Figure 1:**
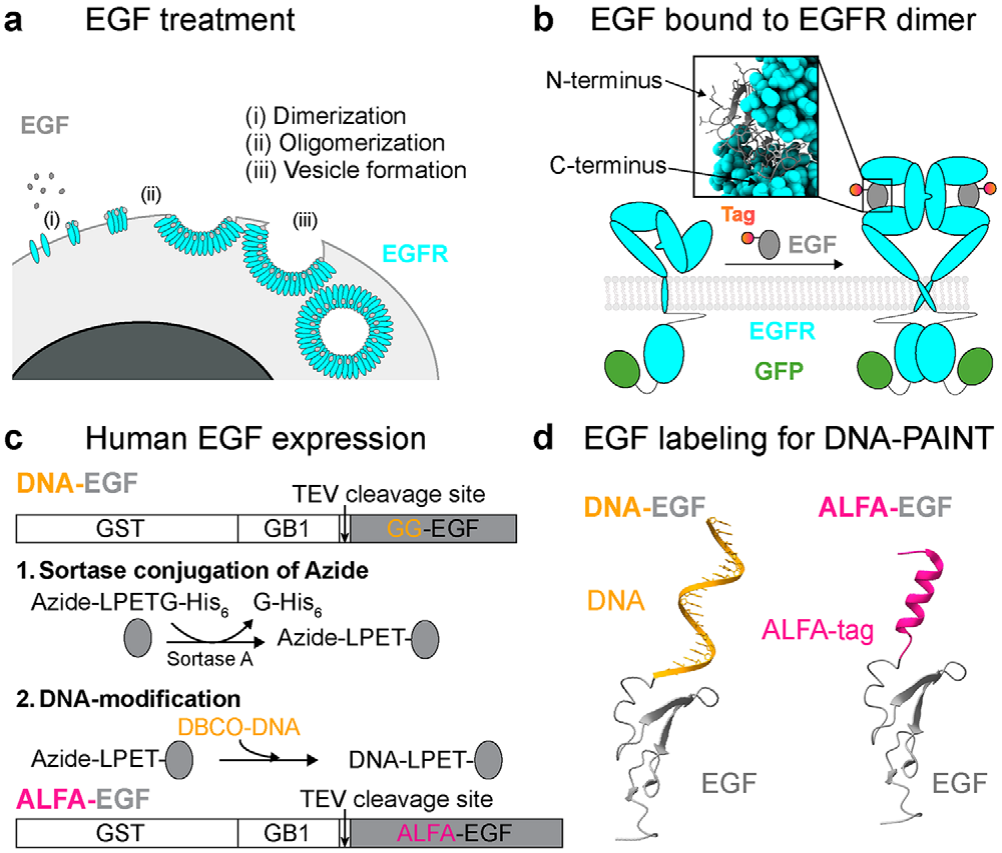
Labeling approaches of EGF. a) EGF treatment promotes receptor clustering through dimerization and oligomerization, eventually resulting in vesicle formation within cells. b) The EGF ligand binds to the EGF receptor (EGFR), inducing EGFR dimerization. Upon binding, the EGF C‐terminus faces EGFR and the EGF N‐terminus is accessible. EGFR is labeled with an intracellular C‐terminal GFP‐tag. c) Expression and purification of EGF involve an N‐terminal GST‐tag for stability and purification and a GB1‐tag to enhance yield. For site‐specific N‐terminal labeling, a glycine‐rich sequence (GG) enables Sortase‐mediated conjugation of EGF with an azide‐modified peptide Azide‐LPETGG‐HHHHHH. This azide‐labeled EGF is subsequently coupled with DBCO‐DNA, yielding DNA‐EGF. An alternative version, ALFA‐EGF, is expressed similarly, incorporating an ALFA‐tag immediately upstream of EGF. d) DNA‐EGF is directly labeled via a DNA conjugate, while ALFA‐EGF features an N‐terminal peptide tag, allowing for subsequent labeling with a DNA‐linked ALFA nanobody.

Labeling of EGF ligands for DNA‐PAINT imaging has to mainly fulfill two requirements: 1) Conjugated EGF has to exhibit similar functionality (i.e., the ability to induce dimerization, activation, and internalization of EGFR) as native unconjugated EGF and 2) the EGF tag has to be fully accessible for DNA‐PAINT imaging. Upon EGF treatment, EGFR oligomerizes, forming higher‐order complexes that are crucial for effective signaling and internalization. The EGFR‐EGF complex is internalized via vesicle formation (**Figure** **1**a). Based on the structure of this EGF‐EGFR complex^[^ ^30^ ^]^ (PDB ID: 3NJP), the C‐terminus of EGF is embedded into the binding pocket of EGFR (Figure 1b, inset). Thus, to minimize interference with the functionality of EGF and to maintain the addressability of EGF for subsequent DNA‐PAINT imaging, we carried out specific tagging at the N‐terminus.

In humans, EGF is first expressed as a single‐pass transmembrane protein (pro‐EGF), which undergoes proteolytic cleavage at the cell surface to release the mature and active N‐terminal ectodomain as soluble EGF.^[^ ^31^ ^]^ Thus, the correct folding of EGF depends on the expression as a fusion protein. To achieve this, we added the N‐terminal B1 domain of *Streptococcal* protein G (GB1) facilitating expression^[^ ^32^ ^]^ and an N‐terminal Glutathione‐S‐Transferase (GST)‐tag, enabling high yield of functional EGF after Tobacco‐Etch Virus (TEV) cleavage (Figure 1c; Figure S1, Supporting Information).

**Figure 2:**
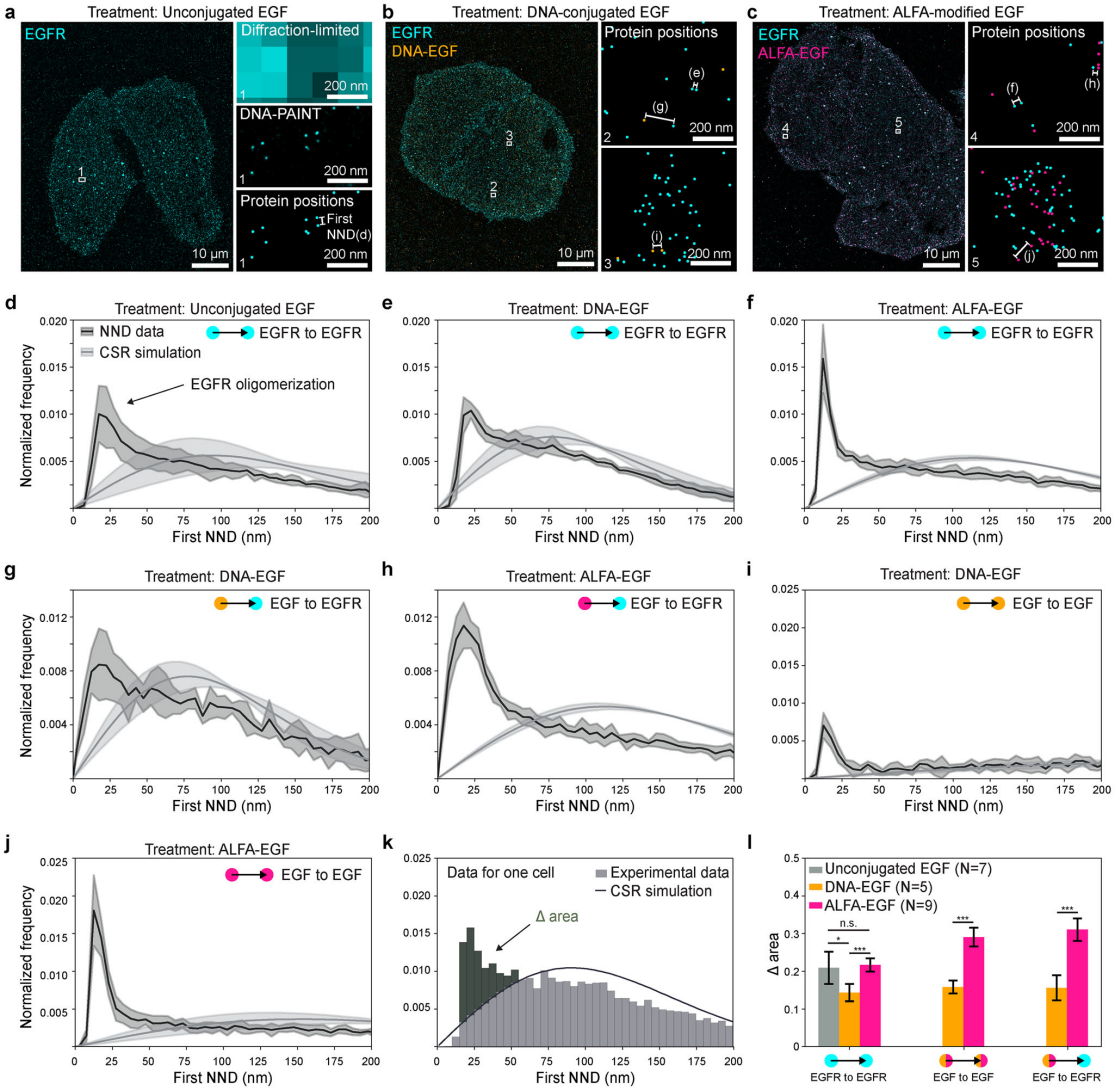
Visualization and functional analysis of DNA‐EGF and ALFA‐EGF labeling in EGFR‐expressing cells. a–c) DNA‐PAINT images of A549 cells expressing EGFR‐mTagGFP2 treated with (a) unconjugated EGF, (b) DNA‐EGF, and (c) ALFA‐EGF. Insets show zoomed views of clustered localizations with protein positions determined by the center of mass of each cluster. DNA‐EGF (b) shows minimal co‐localization with EGFR across areas of varying EGFR density (2 and 3), while ALFA‐EGF (c) exhibits a high degree of co‐localization with EGFR in both low and high EGFR density regions (4 and 5). d–f) EGFR oligomerization was assessed by measuring the first‐nearest neighbor distance (NND) of EGFR positions for cells treated with unconjugated EGF, DNA‐EGF, and ALFA‐EGF. Solid lines represent the mean NND for each treatment (N = 7 for unconjugated EGF, N = 5 for DNA‐EGF, N = 9 for ALFA‐EGF), with the shaded areas indicating standard deviation (STD). A completely spatially random (CSR) distribution is overlaid for comparison. g, h) Specific binding of DNA‐EGF and ALFA‐EGF to EGFR was evaluated through cross‐NND measurements from EGF to EGFR positions, with CSR distributions included for reference. i, j) EGF‐to‐EGF NND histograms reveal a non‐random peak for both DNA‐EGF and ALFA‐EGF compared to CSR, indicating non‐random spatial proximity of the labeled EGF molecules. k) Quantitative analysis of binding interactions was performed by calculating the area above the CSR curve in the normalized NND histograms (Δ area) for experimental data. l) Bar plot comparing the mean values of “Δ areas” from the NND histograms in (d–j), highlighting differences in binding and clustering. The EGFR to EGFR NND shows that DNA‐EGF promotes lower EGFR oligomerization (Δ area: 0.14 ± 0.02) compared to unconjugated EGF (0.21 ± 0.04), while ALFA‐EGF (0.22 ± 0.02) induces a similar extent of EGFR oligomerization as unconjugated EGF. The EGF to EGFR cross‐NND indicates higher colocalization for ALFA‐EGF (Δ area: 0.31 ± 0.03) than DNA‐EGF (0.16 ± 0.03), and EGF to EGF NND analysis demonstrates increased clustering with ALFA‐EGF (Δ area: 0.29 ± 0.03) compared to DNA‐EGF (0.16 ± 0.02), suggesting superior labeling and binding efficiency for ALFA‐EGF. *p*‐values were determined with one‐sided ANOVA testing (^***^: p <  0.001; ^**^: *p* <  0.01; ^*^: *p* <  0.05; n.s.: *p* >  0.05).

We then developed two strategies (Figure 1d) for modifying EGF with DNA: 1) directly labeling EGF with DNA docking strands and 2) using an ALFA tag to which DNA‐conjugated nanobodies could bind after incubation in a cellular context. To site‐specifically add a DNA docking strand using the first strategy, we chose the Sortase system that allows enzyme‐mediated coupling of poly‐Glycine‐tagged proteins to LPXTG‐modified proteins.^[^ ^33^ ^]^ GGSGGG‐EGF (further referred to as GG‐EGF) was first functionalized with Azide via an Azide‐coupled peptide that could, in turn, be reacted with dibenzocyclooctyne (DBCO)‐DNA using copper‐independent click chemistry (Figure 1c; Figure S1, Supporting Information). To test the functionality of our site‐specifically labeled EGF constructs, we used the human cancer cell line A549, stably expressing EGFR‐GFP. After treating with 10 nm unconjugated EGF for 10 min, fixing, and imaging with total internal reflection fluorescence (TIRF) microscopy, we were able to detect EGFR‐clustering in the diffraction‐limited GFP‐channel (Figure S2, Supporting Information). By labeling EGFR with a DNA‐conjugated anti‐GFP‐nanobody and imaging with DNA‐PAINT, we measured EGFR clusters with 200–500 nm in diameter (Figure S2, Supporting Information). Similar to unconjugated EGF, both DNA‐EGF and ALFA‐EGF resulted in the expected EGFR clustering, as shown by diffraction‐limited as well as DNA‐PAINT imaging (**Figure** **2**a–c; Figure S2, Supporting Information). In contrast, no EGFR clustering was detected in untreated cells.

**Figure 3:**
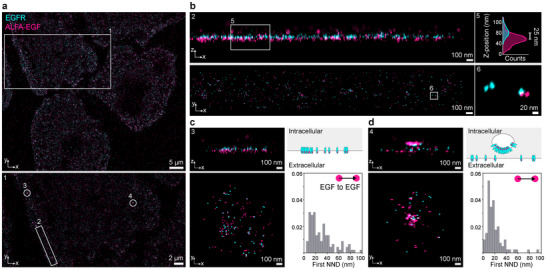
3D visualization of ALFA‐EGF and EGFR interactions in cells. a) 3D DNA‐PAINT image of A549 cells, showing EGFR‐GFP (cyan) and ALFA‐EGF (magenta). The zoomed‐in region (1) highlights clusters of EGFR and ALFA‐EGF on the cell membrane. b) In a homogeneous cell area (zoom‐in of the region (2) from (a, bottom)), the z‐x view reveals two distinct layers: intracellularly‐GFP‐tagged EGFR visualized using GFP‐Nanobodies (cyan) and extracellularly bound ALFA‐EGF (magenta). A cross‐sectional z‐position histogram of area (5) shows a peak‐to‐peak distance of ≈25 nm between EGFR and ALFA‐EGF, indicative of their axial spatial separation. c) In regions with EGFR and ALFA‐EGF clustering (zoom‐in of the region (3) from (a, bottom)), the EGF‐to‐EGF nearest neighbor distance histogram indicates clustering at short distances. d) In more mature vesicles (zoom‐in of region (4) from (a, bottom)), shorter, non‐random EGF‐to‐EGF distances become more prominent, as evidenced by the increased frequency of short NND peaks, suggesting a higher degree of ligand clustering in vesicles.

To qualitatively evaluate the functionality and accessibility of the DNA‐conjugated EGF and the ALFA‐tagged EGF, we performed Exchange‐PAINT experiments.^[^ ^11^ ^]^ The DNA‐conjugated EGF was directly measured with DNA‐PAINT since it already carries the docking strand. The ALFA‐EGF was imaged by incubating DNA‐conjugated anti‐ALFA‐nanobodies on the fixed cell sample. EGFR and EGF were targeted with orthogonal docking strands and image acquisition was carried out using Exchange‐PAINT. Since EGF induces oligomerization of EGFR, we assessed the functionality of the DNA‐EGF and the ALFA‐EGF by comparing their EGF‐induced oligomerization of EGFR to cells treated with unconjugated EGF. For this, we determined the nearest neighbor distances (NND) for the different EGFs across multiple cells. Specifically, we analyzed 1) EGFR‐EGFR NNDs to evaluate the EGF‐induced EGFR oligomerization (Figure 2d–f), 2) EGF‐EGFR cross‐NND to quantify the accessibility of the EGF and thus the co‐localization of EGFR with EGF (Figure 2g,h), and 3) EGF‐EGF NND to further confirm the oligomerization behavior (Figure 2i,j). To further quantify the NND histograms, we simulated complete spatial randomness (CSR) of the measured receptor and ligand densities and calculated the area between the experimental NND plots and the CSR curves (Denoted as “Δ area”, Figure 2k,l).

The EGFR‐EGFR NND analysis revealed that ALFA‐tagged EGF induces EGFR clustering behavior similar to that of unconjugated EGF and demonstrates increased co‐localization with EGFR. This suggests that the ALFA‐tag does not interfere with EGF's binding to the tight binding pocket of EGFR. In contrast, DNA‐conjugated EGF showed reduced oligomerization (indicated by a smaller “Δ area”). The EGF‐EGFR cross‐NND also shows that we detect more co‐localization of ALFA‐EGF with EGFR compared to DNA‐EGF to EGFR. ALFA‐EGF led to more EGFR clustering compared to DNA‐EGF. This is consistent with the apparent increased oligomerization of EGFR induced by ALFA‐EGF, and the specific binding of ALFA‐EGF to EGFR (Figure 2c). These findings indicate that DNA‐conjugated EGF may not be fully functional, as it shows reduced oligomerization compared to unconjugated EGF and lower co‐localization with EGFR. Adding the DNA docking strand to the azide‐EGF after fixation led to a high non‐specific background (Figure S3, Supporting Information), compromising the accuracy of this approach.

Using ALFA‐tagged EGF, we are now able to study the interaction of ligands with their receptors at single‐molecule resolution, gaining more insight into the spatial arrangement of EGFR and EGF. In a 3D measurement, we captured specific distinct stages of receptor internalization on one cell (**Figure** **3**a). Here, we identified two axial layers (Figure 3b): one corresponding to intracellularly visualized EGFR and the other to extracellularly visualized EGF. The measured distance between these layers was ≈25 nm, aligning well with the thickness of the cell membrane when accounting for tag size and label position. We could observe specific phases of receptor internalization (Figure 3c, d), starting with receptor oligomerization and clustering on the membrane and progressing to fully internalized EGFR‐EGF vesicles. These stages are reflected in the EGF‐EGF nearest‐neighbor distance (NND) histograms. As internalization progresses, the peaks in the EGF‐EGF NND histograms shift toward shorter distances, indicating increased proximity.

Notably, we resolved the relative spatial arrangement of EGF and EGFR within these vesicles, with EGF positioned toward the vesicle interior and EGFR oriented outward, aligning with the anticipated internalization process. This ligand labeling protocol for EGF enabled us to directly resolve both ligand‐to‐ligand and ligand‐to‐receptor distances in 3D.

## 3 Conclusion

In this study, we introduced a method for site‐specific labeling of the small extracellular ligand EGF, enabling its visualization using multiplexed DNA‐PAINT super‐resolution microscopy. Our results show that direct conjugation of EGF with DNA reduces its functional ability, partially impairing its capacity to induce EGFR oligomerization compared to the unconjugated ligand. This effect may stem from electrostatic interactions between the negatively charged DNA and EGF, which could hinder the binding or activation of EGFR. Additionally, the DNA‐docking strand attached to EGF might become partially inaccessible when the ligand is bound to its receptor. To overcome these limitations, we employed an alternative labeling strategy using the small ALFA‐tag, which does not affect EGF function. This approach preserved EGF's ability to induce EGFR oligomerization at wild‐type levels (Figure 2). ALFA‐EGF can be incubated with living cells to activate EGFR and then targeted with DNA‐conjugated anti‐ALFA nanobodies after fixation, yielding DNA‐docking strands that are accessible for subsequent DNA‐PAINT imaging. This novel ligand labeling approach enabled us for the first time to directly visualize the relative spatial arrangements of EGF and EGFR at single‐protein resolution using DNA‐PAINT. We found a close proximity of ALFA‐EGF and EGFR (≈17 nm in 2D), as expected from the X‐ray structure (Figure 2). In addition, the specific addressability of ALFA‐EGF enabled us to introduce anti‐ALFA single‐domain antibodies, each labeled with one of three orthogonal DNA sequences, for RESI imaging in 3D. This allowed us to resolve two ALFA‐EGFs bound to the same EGFR dimer with a distance of 11 nm, as predicted by the EGF‐EGFR structure^[^ ^30^ ^]^ (PDB ID: 3NJP) (Figure 3). Furthermore, we were able to visualize downstream steps in EGFR signaling by imaging vesicle formation and internalization after EGF‐induced activation (Figure 3).

A detailed understanding of EGFR oligomerization upon EGF‐mediated activation and their spatial arrangement could guide structure‐specific drug design, by disrupting or enhancing oligomerization and thus modulating EGFR signaling.

We developed a general ligand expression strategy utilizing GST‐ and GB1‐tags for high yields and efficient purification and the ALFA tag for efficient detection. Due to its small size of 14 amino acids and its independent folding, it minimally impacts ligand function. Unlike HA or myc tags, the ALFA‐tag forms a stable alpha‐helix and remains specifically addressable after fixation, making it ideal for super‐resolution microscopy. It can be labeled with a high‐affinity single‐domain antibody (≈40% labeling efficiency in DNA‐PAINT).^[^ ^34^ ^]^ ALFA‐tag's versatility allows placement at the N‐terminus, C‐terminus, or within a protein, enabling broad ligand applications. Beyond super‐resolution microscopy, ALFA‐tagged ligands can be used for immunoprecipitation, immunoblotting, and in vivo ligand detection.^[^ ^19^ ^]^ Expanding orthogonal tag‐binder pairs will enable multiplexed imaging of different ligands. Taken together, our ligand labeling method is a stepping stone to study – with an unprecedented level of detail – ligand‐receptor interactions, offering new insights into complex signaling networks.

## 4 Experimental Section

### Materials

DNA oligonucleotides modified with C3‐azide, Cy3B, and DBCO were ordered from Metabion and MWG Eurofins. Sodium chloride (NaCl; 5 m; AM9760G), potassium chloride (KCl; 2 m; AM9640G), calcium chloride (CaCl₂; 1 m, 15445389), ultrapure water (10977‐035), Tris (1 m, pH 8; AM9855G), ethylenediaminetetraacetic acid (EDTA; 0.5 m, pH 8.0; AM9260G), 1× phosphate‐buffered saline (PBS; pH 7.2; 20012–019), 10× PBS (70011051), fetal bovine serum (FBS; 10500‐064), 0.05% trypsin–EDTA (25300‐ 054), Dubecco's modified eagle medium (DMEM; 61965026) and Salmon Sperm DNA (15632011) were purchased from Thermo Fisher Scientific. Human EGF (E9644‐.2MG), bovine serum albumin (BSA; A4503‐10G), TritonX‐100 (93443), and Millipore Millex 33 mm MCE 0.22 µm sterile filter (SLGS033) were ordered from Sigma–Aldrich. Ammonium chloride (NH_4_Cl; K298.1) was purchased from Carl Roth. Sodium hydroxide (NaOH; 31627.290) was purchased from VWR. Methanol‐free paraformaldehyde (PFA; 15710) was obtained from Electron Microscopy Sciences. Glutaraldehyde (23115.01) was purchased from SERVA. Tween‐20 (P9416‐50ML), glycerol (65516‐500 mL), methanol (32213‐2.5L), protocatechuate 3,4‐dioxygenase *pseudomonas* (PCD; P8279), 3,4‐dihydroxybenzoic acid (PCA; 37580‐25G‐F), (±)‐6‐hydroxy‐2,5,7,8‐tetra‐methylchromane‐2‐carboxylic acid (trolox; 238813–5G), sodium azide (NaN_3_; 769320) and A549 EGFR‐TagGFP2 cells (CLL 1141) were obtained from Sigma–Aldrich. Ninety‐nanometer gold nanoparticles (G‐90‐100) were ordered from Cytodiagnostics. PureCube 100 Ni‐INDIGO Agarose was purchased from Cube Biotech (75103). Anion exchange column RESOURCE Q (17117701) and Glutathione Sepharose 4B (17075605) were obtained from Cytiva. Nanobodies against GFP (clone 1H1, N0305) and against ALFA (N1505) with a single ectopic cysteine at the C‐terminus for site‐specific conjugation were purchased from Nanotag Biotechnologies. DBCO‐PEG4‐Maleimide (CLK‐A108P) was purchased from Jena Bioscience. Microscope slides were obtained from ibidi (µ‐Slides with 8 wells with a glass bottom, 80801).

### Buffers

The following buffers were used for sample preparation and imaging:

i) Imaging buffer: 1×PBS, 1 mm EDTA, 500 mm NaCl (pH 7.4), 0.02% Tween; supplemented with 1×Trolox, 1×PCA and 1×PCD, filtered with 0.2 µm filter
ii) Blocking buffer: 1xPBS, 1 mm EDTA, 0.02% Tween‐20, 0.05% NaN_3_, 2% BSA, 0.05 mg mL^−1^ salmon sperm DNA, filtered with 0.2 µm filter
iii) Quenching buffer: 2 m NH_4_Cl in ultrapure water, filtered with 0.2 µm filter

### Expression and Purification of EGF

GST‐GSGS‐GB1‐TEV‐GG‐EGF or GST‐GSGS‐GB1‐TEV‐ALFA‐GSGS‐EGF were expressed in *Escherichia coli* T7 SHuffle Express strain that enables disulfide bridge formation.^[^ ^35^ ^]^ After expression, cells were harvested and lysed by sonication. The lysate was clarified by centrifugation at 50000 g for 30 mins at 4 °C. For GST Affinity Purification, the clarified lysate was loaded onto a Glutathione Sepharose 4B column equilibrated with Tris‐HCl (50 mm, pH 7.5) and NaCl (200 mm). After washing with 10 column volumes (CV) of binding buffer, elution of the GST fusion protein was performed by His‐tagged TEV protease‐cleavage (1 mg mL^−1^ in 50 mm Tris‐HCl, 200 mm NaCl) of GST‐GSGS‐GB1‐tag and overnight incubation at 4 °C. The cleaved protein was further purified using a Superdex 30 Increase GL 10/300 column equilibrated with Tris‐HCl (50 mm, pH 7.5) and NaCl (150 mm). Fractions containing the purified GG‐EGF or ALFA‐EGF were pooled. ALFA‐EGF was purified in the same way. Protein samples were analyzed by SDS‐PAGE using 4–22% gradient gels under non‐reducing conditions.

### Sortase Conjugation of GG‐EGF

To site‐specifically functionalize GG‐EGF an Azide‐peptide was used. GG‐EGF (100 µm) was reacted with the peptide (pep, Azide‐LPETGG‐HHHHHH) (1 mm), Sortase A (10 µm) in Tris‐HCl (50 mm, pH 7.5), NaCl (150 mm), supplemented with CaCl₂ (10 mm). The reaction was incubated at 4 °C for 3 h. The conjugated product was purified by passing the mixture through Ni‐INDIGO Agarose (200 µL) to remove His‐tagged Sortase A and excess peptide.

### DBCO‐DNA Conjugation of GG‐EGF

Azide‐EGF was further conjugated with DBCO‐7xR3. A reaction mixture of Azide‐EGF (40 µm) was incubated with equimolar amounts of DBCO‐DNA overnight at 4 °C. The conjugated product was purified using a ResourceQ 5/50 column. Elution was performed using a linear gradient of 0–50% Buffer B (1x PBS, 1 m NaCl, pH 7.4). Fractions containing the 7xR3‐EGF conjugate were pooled and concentrated to 250 µL with Amicon Ultra 3 kDA filters (Figure S1, Supporting Information).

### PCA, PCD, and Trolox

A 100× Trolox solution was prepared by dissolving Trolox (100 mg) in 100% methanol (430 µL) and NaOH (1 m, 345 µL) in water (3.2 mL). For the 40× PCA solution, PCA (154 mg) was mixed with water (10 mL), and the pH was adjusted to 9.0 using NaOH. The 100× PCD solution was made by dissolving PCD (9.3 mg) in 13.3 mL of buffer containing Tris‐HCl (100 mm, pH 8), KCl (50 mm), EDTA (1 mm), and glycerol (50%).

### Microscope Setup

Fluorescence imaging was carried out on an inverted microscope (Nikon Instruments, Eclipse Ti2) with the Perfect Focus System, applying an objective‐type TIRF configuration equipped with an oil‐immersion objective (Nikon Instruments, Apo SR TIRF×100, NA 1.49, Oil). A 560‐nm laser (MPB Communications, 1 W) was used for excitation and coupled into the microscope via a Nikon manual TIRF module. The laser beam was passed through a cleanup filter (Chroma Technology, ZET561/10) and coupled into the microscope objective using a beam splitter (Chroma Technology, ZT561rdc). Fluorescence was spectrally filtered with an emission filter (Chroma Technology, ET600/50 m, and ET575lp) and imaged on an sCMOS camera (Hamamatsu Fusion BT) without further magnification, resulting in an effective pixel size of 130 nm (after 2×2 binning). TIR illumination was used for all measurements. The camera's central 1152×1152 pixels (576×576 after binning) were used as the region of interest. Raw microscopy data was acquired using µManager (Version 2.0.1). 3D imaging was performed using a cylindrical lens (Nikon Instruments, N‐STORM) in the detection path.

### Cell Culture

A549 EGFR‐TagGFP2 cells were cultured at 37 °C and 5% CO_2_ in DMEM medium supplemented with 10% FBS. Cells were passaged every 2–3 days using trypsin‐EDTA. mTagGFP2 was further referred to as GFP.

### Nanobody‐DNA Conjugation

The anti‐GFP nanobody and the anti‐ALFA were conjugated to a DBCO‐PEG4‐Maleimide linker. After removing the unreacted linker with Amicon centrifugal filters (10 000 MWCO), the DBCO‐nanobody was conjugated via DBCO‐azide click chemistry to one of the DNA docking strands according to Table S1 (Supporting Information).^[^ ^9^ ^]^

### Cell Sample Preparation

A549 GFP‐EGFR cells (10 000 cm^−2^) were seeded on eight‐well high glass‐bottom chambers. The next day, the cells were washed 3 times with serum‐free DMEM medium and starved for 6h. Afterward, the cells were treated for 10 min with the specifically labeled EGF (10 nm) in a serum‐free DMEM medium. The cells were then fixed with pre‐warmed methanol‐free PFA (4%) in 1xPBS for 15 min. After washing 3 times with 1xPBS, the cells were permeabilized with TritonX‐100 (0.125%) in 1xPBS for 2 min. After washing 3 times with 1xPBS, the cells were blocked with blocking buffer at 4 °C overnight. For EGFR‐GFP imaging 25 nm DNA‐conjugated anti‐GFP and anti‐ALFA nanobodies in blocking buffer were incubated for 1 h at room temperature (RT), while RESI imaging requires R6 anti‐GFP (25 nm) and orthogonally labeled R1‐R3 anti‐ALFA nanobodies (6.25 nm each) in blocking buffer were incubated overnight at 4 °C (for DNA sequences see Table S1, Supporting Information). The cells were washed 3 times with 1xPBS, post‐fixed with PFA (4%) and glutaraldehyde (0.2%) in 1xPBS for 10 min. Then, the cells were quenched with freshly prepared NH_4_Cl (200 mm) in 1xPBS from the quenching buffer for 5 min and washed 3 times with 1xPBS. 90 nm gold nanoparticles in 1:1 in 1xPBS were incubated for 5 min at RT. The cells were washed 3 times with 1xPBS

### EGFR‐GFP Imaging

The samples were imaged in an imaging buffer with 200 pm imager strand (sequences see Table S1, Supporting Information), for 40 000 frames with 100 ms exposure time per frame at 30 mW laser power after the objective, corresponding to a power density of 150 W cm^−2^.

### RESI 3D Imaging

The samples were imaged in an imaging buffer with 200 pm imager strands (for DNA sequences see Table S1, Supporting Information), for 40 000 frames with 100 ms exposure time per frame at 30 mW laser power after the objective, corresponding to a power density of 150 W cm^−2^. In between every imaging round, the current imager strands were removed by washing the sample with 1xPBS until no blinking events were observed on the microscope.

### Statistical Analysis

The raw fluorescence single‐molecule localization data were processed for super‐resolution reconstruction using the Picasso software package^[^ ^8^ ^]^ (the latest version is available at https://github.com/jungmannlab/picasso). For this, single molecules were localized in Picasso using the Gaussian least squares option. The z coordinate of 3D measurements was determined using a 3D calibration file, based on the degree of astigmatism.^[^ ^36^ ^]^ Drift correction was performed using a redundant cross‐correlation method with gold particles serving as fiducials. For measurement with multiple imaging rounds, all resulting channels were aligned with each other. After that, the protein positions were determined with a clustering algorithm for each channel individually. For this, circular clusters of localizations centered around local maxima were identified and grouped. The centers of the localization groups were calculated as a weighted mean, using the squared inverse localization precisions as the weights, as previously described.^[^ ^13^ ^]^ RESI measurements of the ALFA‐EGF were analyzed as described before.^[^ ^13^ ^]^ The first nearest neighbor distances (NND) of the cluster centers were determined using the Python module Scikit Learn.^[^ ^37^ ^]^ A set of molecules with complete spatial randomness (CSR) distribution was simulated using a custom Python script. For each dataset, the positions of 5 000 000 molecules were simulated based on the measured protein densities. For the analysis in Figure 2d–j, the normalized NND histograms of the experimental and simulated data (with a bin size of 5 nm) for each EGF were summarized by calculating the average (black line) and standard deviation (grey area) of the bar heights across multiple datasets (*N* = 7 for unconjugated EGF, *N* = 5 for DNA‐EGF, *N* = 9 for ALFA‐EGF). The binding interactions were estimated for each dataset by the area (Δ area) of the NND histogram that exceeded the CSR simulation. For this, the normalized histograms of both experimental and simulated data were generated using the same bin size. The positive differences between the simulated and experimental data for NNDs from 0 to 200 nm were summed and multiplied by the bin size to obtain the Δ area. The average and standard deviation of Δ areas of multiple cells were then calculated for each EGF and are shown as a bar graph in Figure 2l. To test the difference of the means of the Δ areas for statistical significance, a one‐sided ANOVA test was performed with the python package scipy^[^ ^38^ ^]^ (^***^: *p* <  0.001; ^**^: *p* <  0.01; ^*^: *p* <  0.05; n.s.: *p* >  0.05).

## Conflict of Interest

The authors declare no conflict of interest.

## Author Contributions

M.H., I.P., and L.H. contributed equally to this work. M.H. designed and analyzed cell experiments, interpreted data, and wrote the manuscript. I.P. designed, performed, and analyzed the conjugation and purification of DNA‐EGF and ALFA‐EGF, interpreted data, and wrote the manuscript. L.H. performed and analyzed experiments and wrote the manuscript. L.A.M. interpreted data, provided input on the data analysis, and wrote the manuscript. L.B. designed the expression and purification strategy for EGF. J.K. and A.P. prepared samples. B.S. supervised the study. R.J. conceived and supervised the study, interpreted data, and wrote the manuscript. All authors reviewed and approved the manuscript.

## Supporting information



Supporting Information

## Data Availability

The data that support the findings of this study are available from the corresponding author upon reasonable request.
