# Supplementary material for: Imaging Ligand‐Receptor Interactions at Single‐Protein Resolution with DNA‐PAINT
Source: Small Methods. 2025 Apr 3;9(6):2401799. doi: 10.1002/smtd.202401799 (PMC12182887; doi:10.1002/smtd.202401799)
Supplement: Supplementary file 1 — Supporting Information [file SMTD-9-2401799-s001.docx]

Supporting Information

Imaging ligand-receptor interactions at single-protein resolution with DNA-PAINT

Monique Honsa, Isabelle Pachmayr, Larissa Heinze, Levent Bas, Luciano A. Masullo, Jisoo Kwon, Ana Perovic, Brenda Schulman, Ralf Jungmann*

**
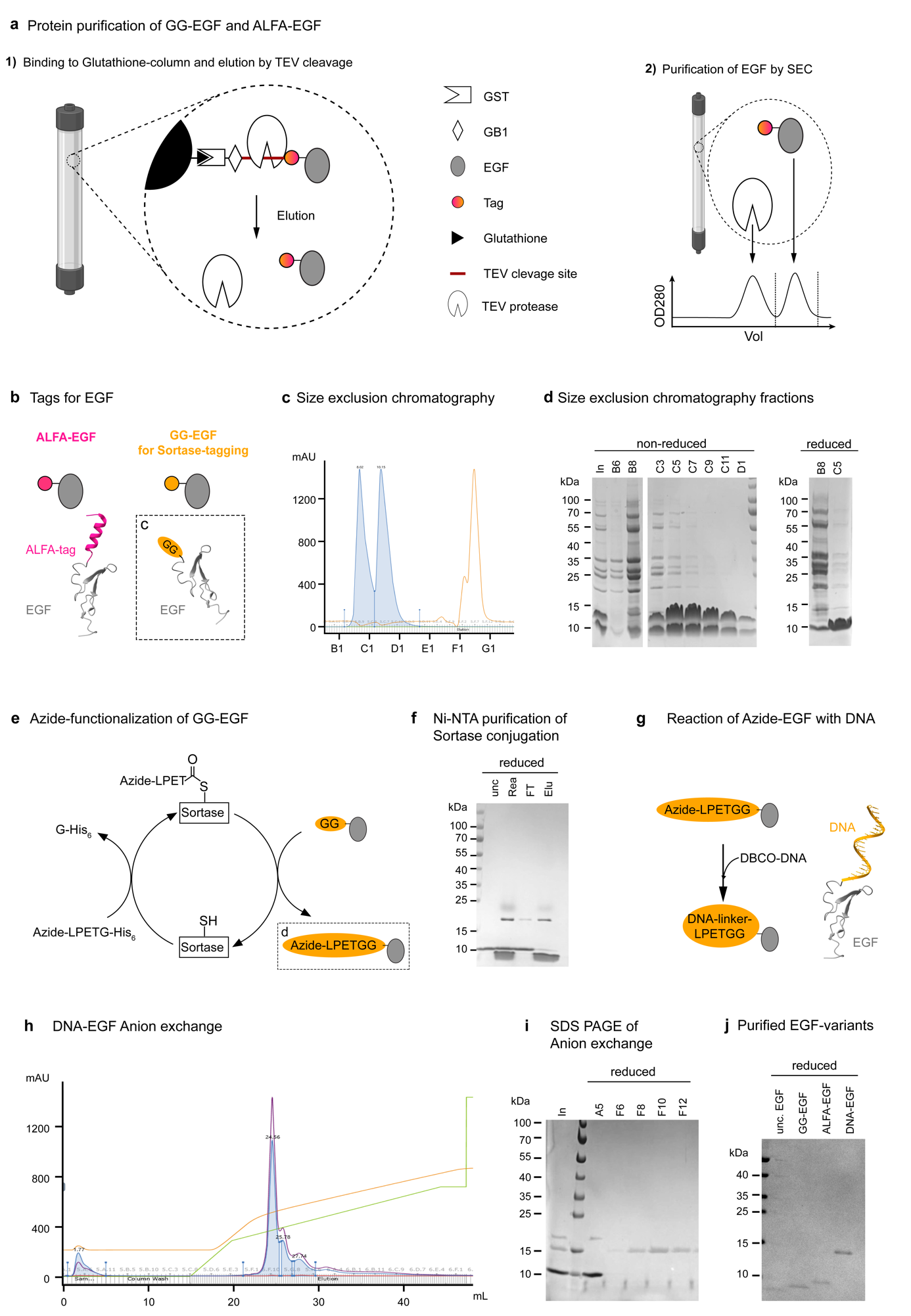
**

**Figure S1 | Purification, tagging, and functionalization of EGF for applications in DNA-PAINT imaging.** **(a)** Purification of glycine-glycine-tagged EGF (GG-EGF) GG-EGF and ALFA-EGF by affinity purification and size exclusion chromatography. The GST-GB1-tag-EGF fusion protein binds to a glutathione column via the GST tag. A Tobacco-Etch Virus (TEV) protease cleavage site in between GB1 and the tag allows for on-column cleavage and elution of tag-EGF (1). Tag-EGF is further purified and separated from the TEV protease using size-exclusion chromatography (2). **(b)** EGF tagging strategies, including ALFA-tagged EGF (ALFA-EGF) and glycine-glycine-tagged EGF (GG-EGF) for sortase-mediated functionalization. **(c)** Size exclusion chromatography (SEC) of GG-EGF after TEV cleavage. Fractions C5-C12 were pooled.  **(d)** SEC fractions of GG-EGF were analyzed on an SDS-PAGE gel. There are two bands due to variations in disulfide bridge formation. When analyzing GG-EGF on a reducing SDS-PAGE gel, only one band corresponding to the expected size of EGF is obtained. **(e)** Azide-functionalization of GG-EGF using sortase-mediated ligation, enabling site-specific conjugation of an azide-modified LPETGG peptide. **(f)** Ni-NTA based purification of the Sortase-mediated conjugation of GG-EGF. Unconjugated EGF (unc), the reaction mix (Rea), the flow through (FT, containing DBCO-conjugated EGF) and the Elution (Elu, containing His-tagged Sortase and His-tagged peptide) were analyzed on a reducing SDS-PAGE. **(g)** Bioorthogonal conjugation of Azide-EGF with DNA using a strain-promoted azide-alkyne cycloaddition reaction with Dibenzocyclooctyne (DBCO)-functionalized DNA. **(h)** DNA-conjugated EGF was separated from unconjugated EGF (in the flow-through) by Anion-exchange chromatography. **(i)** Reducing SDS-PAGE of anion exchange chromatography of DNA-EGF. Fractions F6-G1 were pooled. **(j)** Reducing SDS-PAGE of unconjugated EGF, GG-EGF, ALFA-EGF and DNA-EGF.


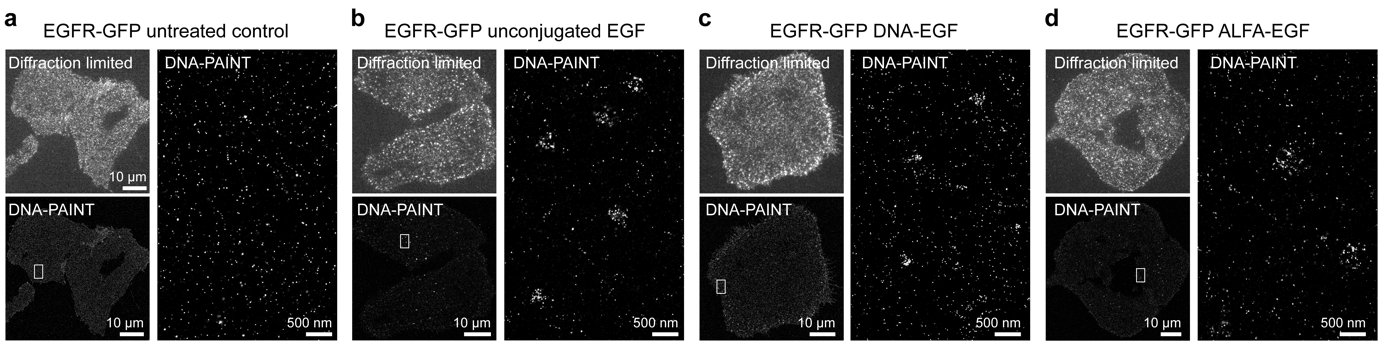


**Figure S2 | Diffraction-limited and DNA-PAINT images of EGFR (untreated and EGF-treated). (a)** Diffraction-limited and DNA-PAINT imaging of untreated EGFR-GFP shows homogeneous distribution of EGFR with no vesicles. **(b)** Diffraction-limited and DNA-PAINT imaging of EGF-treated EGFR-GFP shows vesicle formation of EGFR with ~500 nm in diameter. **(c)** Diffraction-limited and DNA-PAINT imaging of DNA-EGF-treated EGFR-GFP shows vesicle formation of EGFR with ~300 nm in diameter. **(d)** Diffraction-limited and DNA-PAINT imaging of ALFA-EGF-treated EGFR-GFP shows vesicle formation of EGFR with ~500 nm in diameter.


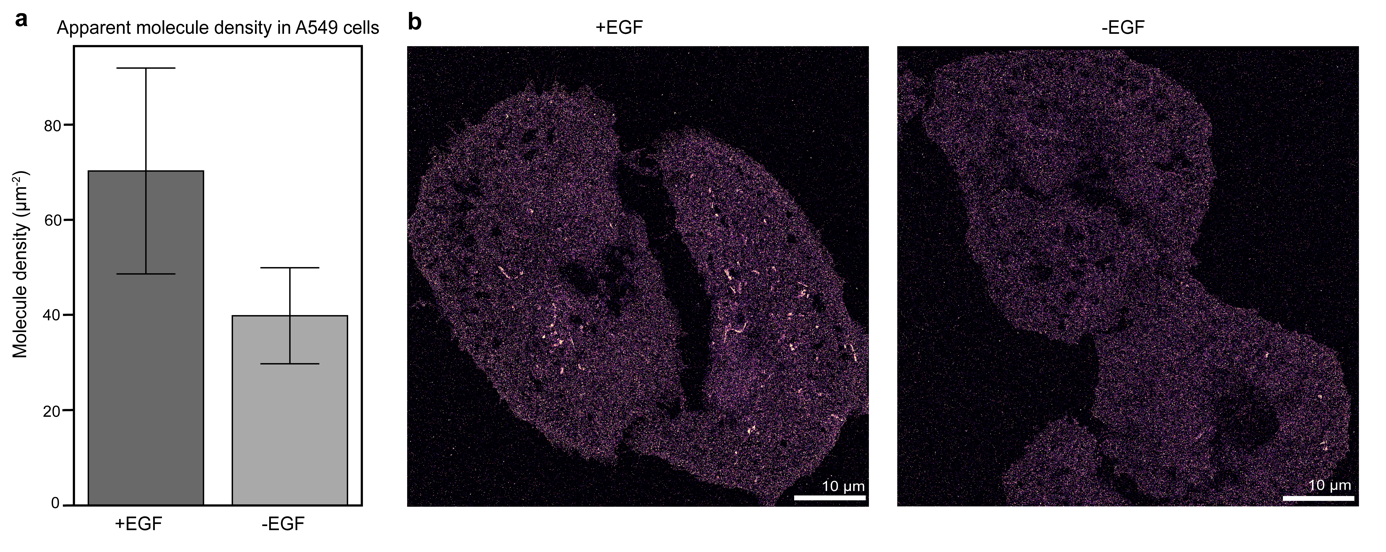


**Figure S3 | DNA-PAINT images of DBCO-conjugated DNA chemically linked “clicked” to azide-EGF (untreated and EGF-treated) after cell fixation. (a)** Measured DBCO-DNA density on A549 cells with DNA-PAINT under two conditions: Azide-EGF-treated (70 ± 22 μm^-2^) and untreated (40 ± 10 μm^-2^). Bars represent the mean and error bars indicate standard deviation (STD). N=8 (for each condition). **(b)** Representative DNA-PAINT images of the azide-EGF treated and untreated cases showing clear localizations in both, which indicates nonspecific binding of the DBCO-DNA to the cell. For the measurements, the cells were seeded, starved in serum- free medium for 6 h and then, depending on the condition, treated with 10 nM azide-EGF for 10 min. The cells were then fixed with 4% PFA for 15 min, permeabilized with 0.125% Triton-X for 2 min and blocked with an azide-free blocking buffer overnight at 4°C. DBCO click chemistry was performed by incubating 1μM DBCO-conjugated DNA on the sample for 3 hr at RT.

**Supplementary Table S1 | Strand sequence of docking strands and imagers.**

| **Sequence name** | **Docking site strand sequence (5’ to 3’)** | **Imager strand sequence (5’ to 3’)** |
| --- | --- | --- |
| R1 | TCCTCCTCCTCCTCCTCCT | AGGAGGA-Cy3B |
| R2 | ACCACCACCACCACCACCA | GGTGGT-Cy3B |
| R3 | CTCTCTCTCTCTCTCTCTC | GAGAGAG-Cy3B |
| R4 | ACACACACACACACACACA | GTGTGT-Cy3B |
| R5 | CTTCTTCTTCTTCTTCTTC | GAAGAAG-Cy3B |
| R6 | AACAACAACAACAACAACAA | TGTTGTT-Cy3B |
